# Antimicrobial effect of insect chitosan on *Salmonella* Typhimurium, *Escherichia coli* O157:H7 and *Listeria monocytogenes* survival

**DOI:** 10.1371/journal.pone.0244153

**Published:** 2020-12-22

**Authors:** Diana Ibañez-Peinado, Maria Ubeda-Manzanaro, Antonio Martínez, Dolores Rodrigo

**Affiliations:** Department of Preservation and Food Safety, Instituto de Agroquímica y Tecnología de Alimentos (IATA-CSIC), Paterna, Valencia, Spain; University of Messina, ITALY

## Abstract

The antimicrobial capability of chitosan from *Tenebrio molitor* as compared with chitosan from crustacean (*Penaeus monodon*) on different pathogenic microorganisms of concern in food safety was studied. The antimicrobial effect was tested at pH 5 and pH 6.2 and at two different initial concentrations (10^3^ or 10^6^ CFU/mL). Results indicated that chitosan from both sources have antimicrobial activity, although the effect depended on the microorganism considered (*Salmonella* Typhimurium, *Listeria monocytogenes* and *Escherichia coli* O157:H7). Our results indicated that *Salmonella* was the most resistant bacteria, and that chitosan from insect was less active than chitosan from crustacean, especially against *Salmonella*. Another important factor on antimicrobial activity was the pH of the sample. When chitosan was added to a solution with a pH of 6.2 it was more active against *Listeria* and *Escherichia coli*, than at pH 5.00. Besides, the effect of chitosan appears to decrease with the incubation time, since some increases in counts were observed on *E*. *coli* and *Salmonella* after the 24 and 49 hours of incubation.

## Introduction

Chitosan is a natural and biodegradable biopolymer that has been used in different industrial applications as flocculating and chelating agent, permeability control agent or antimicrobial substance, among others [1–3]. Today, chitosan is mainly produced on an industrial scale from deacetylation of chitin, chis is present in the exoskeleton of crustaceans and insects and in the cell walls of most fungi and some algae [4]. Although residues from crab, prawns, crayfish and shrimps are the main source of chitin [4], the importance of insect chitosan is due to the role that insects play as a sustainable source of protein. Insects are seen as an alternative to the proteins that are conventionally consumed and that come mainly from traditional livestock (cows, chickens or pigs fundamentally) and fish. Besides, the use of insect as protein source will produce two by-side products of undoubted industrial interest, lipids (30–40% total dry weight) [5], that could be used as biofuel and a residual material composed fundamentally by chitin which have some bioactive properties and from which chitosan can be obtained.

Chitin and chitosan have interesting physicochemical, biological and mechanical properties [6]. One of those properties of chitosan is related with its antimicrobial activity. There are diverse studies in which the antimicrobial and antifungal properties of chitosan and several of its derivatives have been revealed [7, 8]. More recently, the effect of the physical form of chitosan on its antibacterial activity against pathogenic bacteria has been studied [9]. Serio et al. [10] studied the chitosan coating as inhibitor of *Listeria monocytogenes* (*L*. *monocytogenes*) on vacuum-packed pork loins and Brown et al. [11] on fresh cheese. It has been also reported that the antibacterial action is usually rapid and eliminates bacteria in few hours [12].

As far as physical properties of chitosan, they are governed, fundamentally, by two factors; the degree of deacetylation and the molecular weight. Moreover, the natural origin as well as the variability on their chemical structure can affect the properties of chitosan and could impact on their industrial utilization [13]. Some studies have revealed that the degree of deacetylation has been associated with the antimicrobial activity of chitosan [14].

Pathogenic microorganisms as *Listeria monocytogenes*, *Salmonella* Typhimurium or *Escherichia coli* (*E*. *coli*) O157:H7 are of concern under the point of view of food safety. Those microorganisms are present in a broad range of foodstuffs contributing every year to a huge amount of food transmitted illnesses. In 2018 (the latest year for which EU data is available), a total of 5.146 disease outbreaks were identified, with bacteria and their toxins being the main causative agent. *Campylobacter*, *Salmonella* spp., *Escherichia coli* and *Listeria monocytogenes* are among the main pathogens linked to foodborne outbreaks in Europe (2011–2020) [15]. *Salmonella enterica* is generally acquired from contaminated food and is a common cause of human gastroenteritis and bacteremia worldwide [15–17]. The common reservoir of *Salmonella* is the intestinal tract of a wide range of domestic and wild animals, which results in a variety of foodstuffs covering both food of animal and plant origin as sources of infection. Transmission often occurs when organisms are introduced in food preparation areas and are allowed to multiply in food, e.g., owing to inadequate storage temperatures, inadequate cooking, or cross-contamination of ready-to-eat (RTE) food [18, 19]. *Salmonella enterica* subsp. *enterica* serovar Typhimurium (*S*. Typhimurium) is one of the most common serovars associated with clinically reported salmonellosis in humans, accounting for at least 15% of infections [20].

*E*. *coli* O157:H7 produces verotoxins. This bacterium is the major serotype that was recognized as a cause of human illness, but not in cattle, its primary host [21, 22]. The source of infection is the contamination of food such as raw or undercooked meat products and raw milk by human and animal feces [23]. The most common sources of Shiga toxin-producing *E*. *coli* (STEC) O157 infection are beef and leafy vegetables [24], but fresh-pressed apple juice or cider, yoghurt, cheese, salad vegetables, and cooked maize have also been implicated [25].

*L*. *monocytogenes* is an opportunistic pathogen that has been recognized as an important foodborne pathogen since the early 1980s [26]. It is resistant to diverse environmental conditions and can grow at temperatures as low as 3°C [27, 28]. *L*. *monocytogenes* can cause invasive disease in livestock, mainly sheep, goats, and cattle [29]. It is found in a wide variety of raw and processed foods such as milk and cheeses, meat (including poultry) and meat products, and seafood and fish products where it can survive and multiply rapidly during storage [30, 31].

Therefore, control measures should be taken to avoid foodborne outbreaks. Synthetic antimicrobials are each time more refused by the consumer worried by the toxicity of those products. In consequence, people are looking for new natural antimicrobials to help in controlling those pathogenic microorganisms. The control of those microorganisms can be carried out by using natural antimicrobials as chitosan to be used in different foodstuffs reducing in this way the annual cases of illnesses transmitted by foods [32–34].

Considering the previously exposed antecedents, a comparative study of the antimicrobial activity of chitosan from *Tenebrio molitor* as an alternative to crustacean chitosan at different pH levels against *E*. *coli*, *L*. *monocytogenes* and *Salmonella enterica* subsp. *Enterica* serovar Typhimurium at different inoculation sizes was carried out.

## Materials and methods

### Microbial strains

Pure cultures of *Listeria monocytogenes* serovar 4b (Spanish Type *Culture Collection* (CECT) 4032) (*L*. *monocytogenes*), *Escherichia coli* O157:H7 (CECT 5947) (*E*. *coli*) and *Salmonella enterica* serovar Typhimurium (CECT 443) (*S*. Typhimurium) were provided by the Spanish Type Culture Collection. Following the procedure described by Sanz-Puig et al. [35], lyophilized samples (0.5 g lyophil) were rehydrated in Tryptone Soy Broth (TSB) (Scharlab S.A., Barcelona, Spain) (*L*. *monocytogenes* and *S*. Typhimurium) or in Luria Broth (LB) (Scharlab S.A., Barcelona, Spain) (*E*. *coli*). Rehydrated cultures were transferred to 500 mL of its corresponding media and incubated in a bath shaker (200 rpm) for 14 h at 37°C. After centrifugation process, glycerinated bacteria cells were maintained in frozen cryovials at -80°C. The final inoculums concentration was determined by plate count and was of 10^8^−10^9^ colony forming units/mL (CFU/mL).

### Chitosan samples preparation

Crustacean chitosan (chitosan from shrimp shells, practical grade) was purchased from Merck (MerckKGaA, Darmstadt, Germany) and insect chitosan (*Tenebrio molitor*) was acquired from MealFood Europe S.L. (Doñinos de Salamanca, Salamanca, Spain). For both chitosans, a 1% (w/v) chitosan sample stock solution was prepared in a 1% (v/v) acetic acid solution. Chitosan solutions at 0.15% (w/v) were prepared from the 1% (w/v) chitosan stock solution (crustacean or insect) in 1% (v/v) acetic acid. Each stock solution was sterilized by 0.45 μm pore size membrane filters.

### Evaluation of antimicrobial activity of chitosan

The antimicrobial activity was tested at pH 5 and pH 6.2 by acidifying with acetic acid (1% w/v) LB for *E*. *coli* and TSB for *L*. *monocytogenes* and *S*. Typhimurium studies and at two different initial concentrations, 10^3^ or 10^6^ CFU/mL (3.2x10^3^ and 2.7x10^6^ CFU/mL, respectively). Microbial growth at these conditions was compared with the one of control samples (without chitosan). Acetic acid controls (LB and TSB at pH 5 and pH 6.2) were also tested to evaluate the possible antimicrobial effect of acidification.

In order to carry out the experiment, each microorganism maintained in cryovials at -80°C was thawed and serial dilutions in peptone water (0.1% (w/v)) were performed until the desired initial inoculum concentration was achieved (10^3^ or 10^6^ CFU/mL). Inoculated media (LB or TSB) were incubated in a bath shaker at 37° C for 49h and sampled at t0 = 0h, t1 = 3h, t2 = 6h, t3 = 8h, t4 = 24h and t5 = 49h. Samples removed at each time were serially diluted in peptone water (0.1%), plated by duplicate and incubated in Luria Agar (LA) (*E*. *coli*) or Tryptone Soy Agar (TSA) (*S*. Typhimurium and *L*. *monocytogenes*) at 37°C for 24h (*E*. *coli* and *S*. Typhimurium) or 48h (*L*. *monocytogenes*). After the incubation period, bacteria cells were counted (CFU/mL). Experimental results were shown as log10 of survival fraction (log S) calculated as (Eq 1):

$$\text{log}\;S=-{Log}_{10}\;(\frac{N}{N_{0}})$$ (1)

where N is bacterial concentration (CFU/mL) at time t (h) and N_0_ initial bacterial concentration (CFU/mL) (t_0_). Therefore, positive values indicate microbial growth and negative values microbial inactivation respect the initial inoculation value (N_0_).

### Statistical analysis

For each condition, three independent repetitions were performed and for each sampling time, two duplicates were taken, plating each one in duplicate (4 plates/sampling time). The analysis of experimental data was carried out by using Statgraphics Centurion XVI (Statpoint Technologies, Inc., USA). Outliers were identified and removed prior data analysis. The statistical significance of data was determined by an Analysis of Variance (ANOVA) (p < 0.05) and differences between groups were determined by Tukey test.

## Results

### Factors affecting antimicrobial activity of chitosan solutions

**Fig 1:**
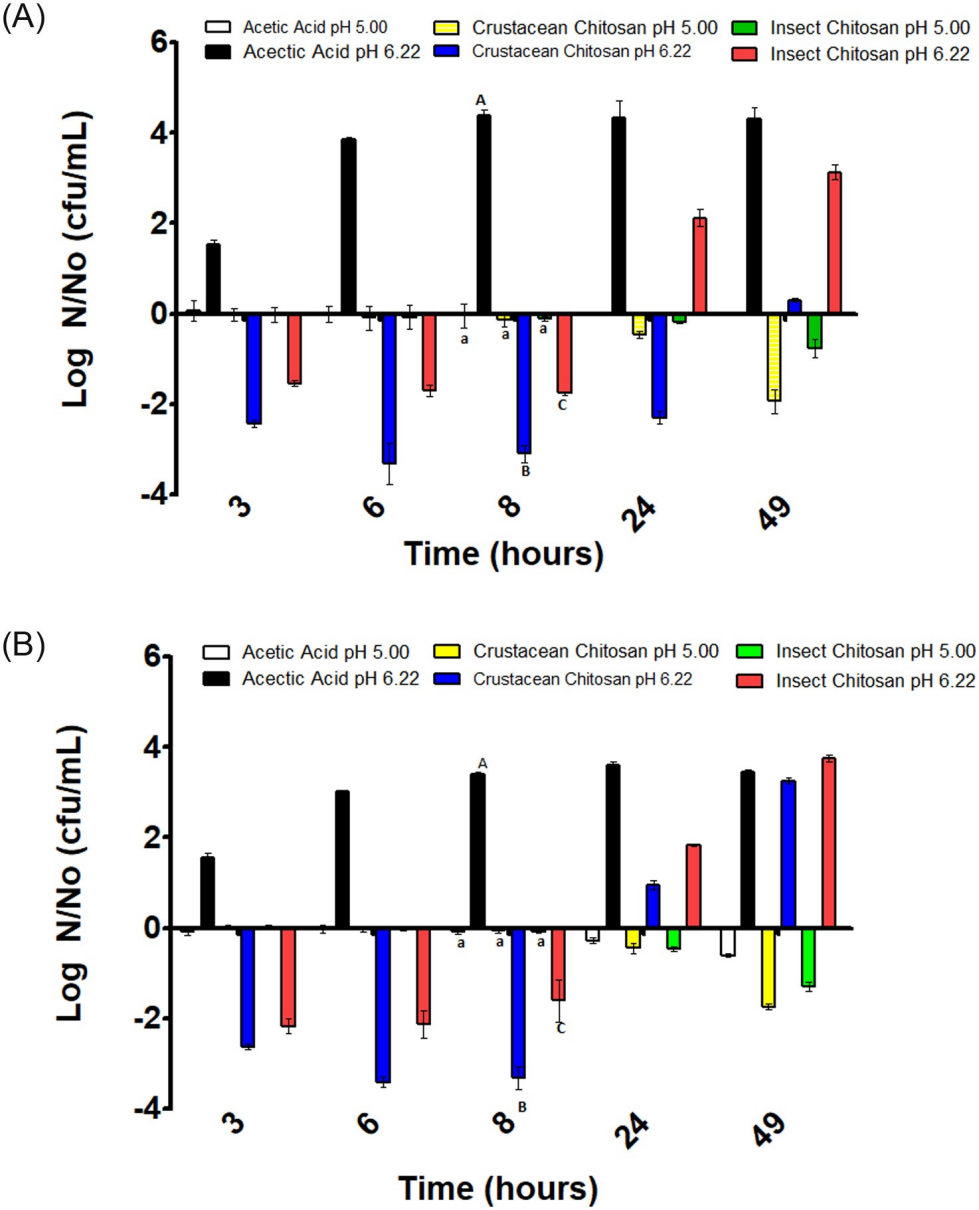
Variation of *E*. *coli* concentration in substrates inoculated with 10^3^ CFU/mL (A) and 10^6^ CFU/mL (B). Positive values indicate growth and negative values inactivation respect the initial inoculation value (N_0_). Different letters mean significant differences between samples of the same pH.

**Fig 2:**
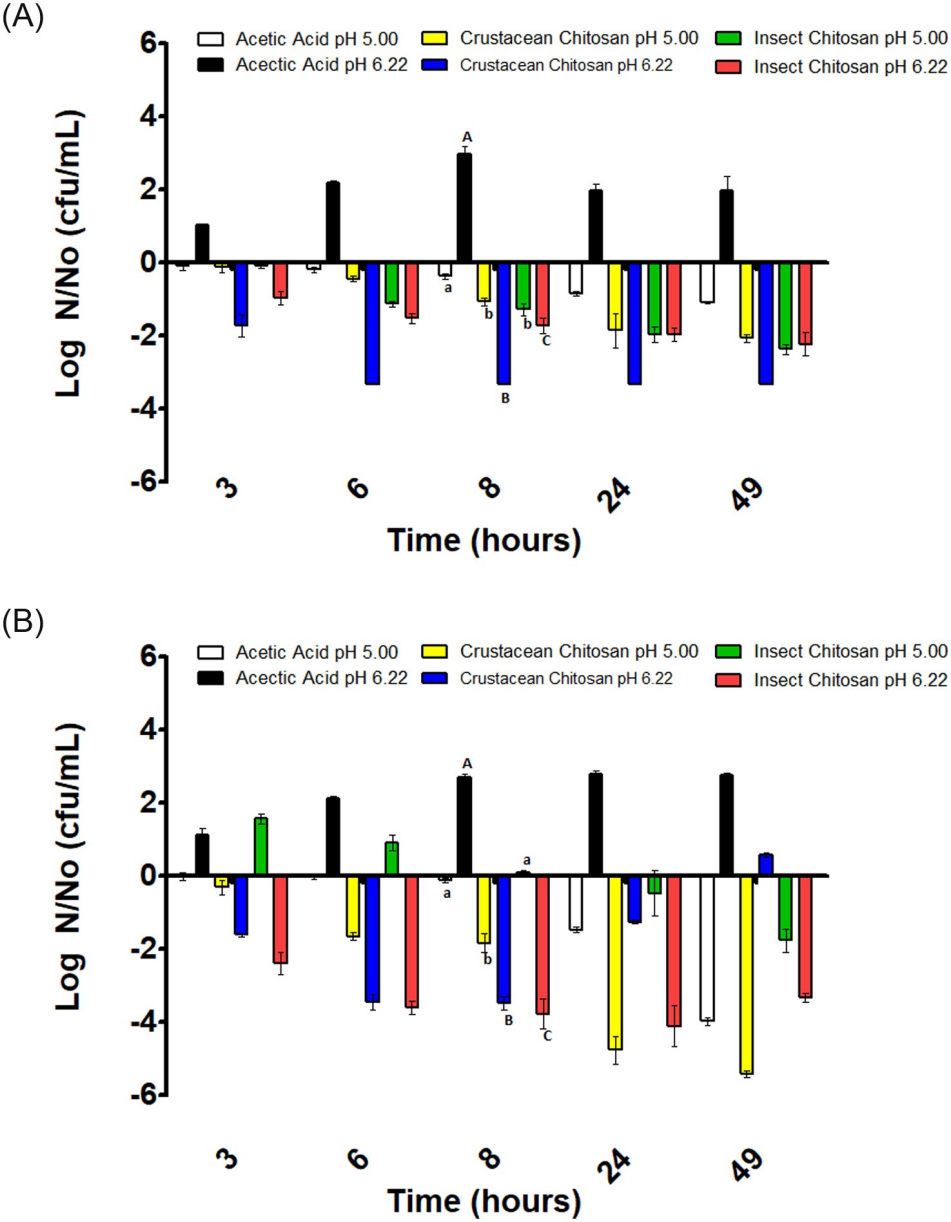
Variation of *L*. *monocytogenes* concentration in substrates inoculated with 10^3^ CFU/mL (A) and 10^6^ CFU/mL (B). Positive values indicate growth and negatives inactivation respect the initial inoculation value (N_0_). Different letters mean significant differences between samples of the same pH.

**Fig 3:**
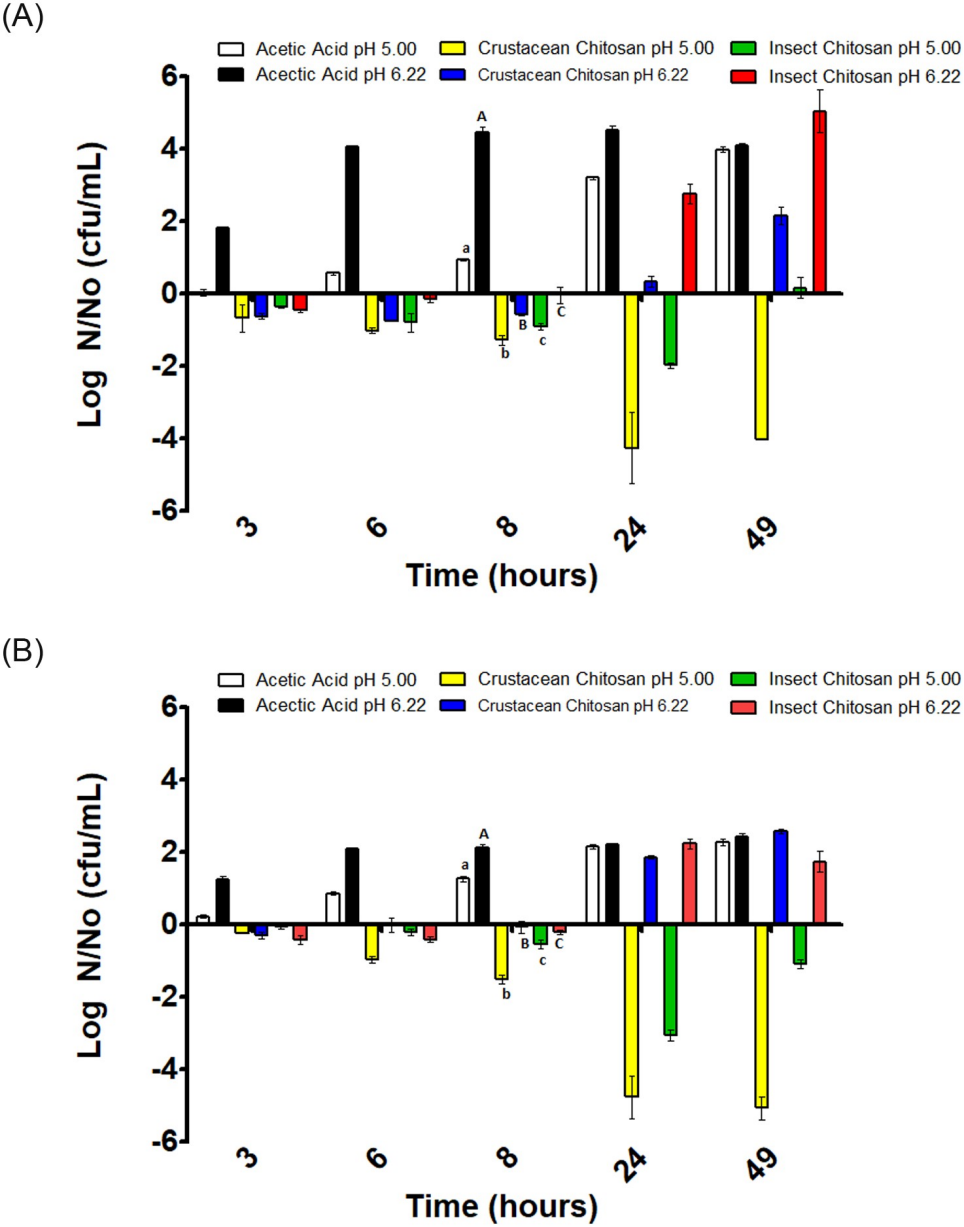
Variation of *S*. Typhimurium concentration in substrates inoculated with 10^3^ CFU/mL (A) and 10^6^ CFU/mL (B). Positive values indicate growth and negative values inactivation respect the initial inoculation value (N_0_). Different letters mean significant differences between samples of the same pH.

Results on the effect of acetic acid and chitosan from different sources on the behavior of *E*. *coli*, *L*. *monocytogenes* and *S*. Typhimurium can be seen in Figs 1–3. In general, the response of tested microorganisms to the different antimicrobials and pH conditions was microorganism dependent. To determine the possible antimicrobial effect of chitosan from different sources dissolved in acetic acid, acetic acid control solutions at pH 5 and 6.2 were tested as antimicrobials for comparison purposes and to define the impact of chitosan independently of the antimicrobial activity of acetic acid used to dissolve chitosan.

### Antimicrobial effects of chitosan solutions on *E*. *coli*

For *E*. *coli* studies with an initial contamination level of 10^3^ CFU/mL, the acetic acid solution at pH 5 resulted in a non-substantial variation on microbial counts during the whole incubation period, 49 h (Fig 1A). A similar result was observed when the inoculum size was 10^6^ CFU/mL (Fig 1B). As for pH 6.2, *E*. *coli* cells grew until reach a plateau at the 8 hour of incubation; probably due to the pH 6.2 represent a more favorable environment for the microorganism (Fig 1A). For an initial inoculum size of 10^6^ CFU/mL the behavior of cells was similar than that at the lower initial microbial concentration. Cells grew until reach the stationary phase at the 8 hours of incubation (Fig 1B).

Regarding the effect of chitosan from crustacean and insect sources at pH 5 against *E*. *coli*, non-appreciable variation on the microbial counts was observed along the incubation period, except at the 49 hours of incubation a light reduction on the microbial counts was observed, being that reduction highest for crustacean chitosan than for insect chitosan (Fig 1A and 1B). Although, this reduction could be due to the decline phase after a stationary phase and not to the presence of chitosan. As for the effect at pH 6.2, the addition of chitosan produced a decrease in the number of cells that was highest in the case of chitosan from crustacean than from insect. However, after 8 hours of incubation, an increase in the microbial counts was observed until the 49 hours of incubation, being the level of microbial concentration higher than one at time zero.

In general, considering both pH levels, the behavior of *E*. *coli* was independently of the initial inoculum size. In consequence, it can be considered that the inoculum size did not affect the microbial pattern along the incubation time.

### Antimicrobial effects of chitosan solutions on *L*. *monocytogenes*

Results for *L*. *monocytogenes* can be seen in Fig 2A and 2B for a 10^3^ and 10^6^ CFU/mL initial concentration level, respectively.

Acetic acid solution at pH 5 produced a decrease in the microbial counts during the incubation period regardless the initial concentration level of *L*. *monocytogenes*. Nevertheless, the number of log reductions was higher when the inoculum size was 10^6^ CFU/mL (Fig 2A and 2B). This result appears to indicate that there is a clear bactericidal effect of the acetic acid against this microorganism. On the other hand, when *L*. *monocytogenes* cells were treated with acetic acid at a pH of 6.2, cells grew reaching the stationary phase after 8 hours of incubation. Results indicated that no bactericidal effect of acetic acid at pH 6.2 took place as compared with the bactericidal effect observed at pH 5.

When *L*. *monocytogenes* cells were subjected to the action of crustacean chitosan in acetic acid at pH 5, a decrease in bacterial counts was observed throughout the incubation period with respect to the initial inoculum, regardless of inoculum size. Nevertheless, the inactivation effect was stronger for the inoculum size of 10^6^ CFU/mL, and no viable cells were observed at the incubation time of 49 hours (Fig 2B).

The effect of crustacean chitosan in acetic acid at pH 6.2 can also be observed in Fig 2A and 2B. For the inoculum size of 10^3^ CFU/mL a rapid reduction in the number of viable cells was observed such that at the 6 hour of incubation no viable cells were observed in the culture. This decrease in cells counts was highest than in acetic acid alone at pH 5. For the inoculum size of 10^6^ CFU/mL, the effect was similar although there is a slight decreasing of the antimicrobial effect that produced a small growth in the number of cells at the 49 hour of incubation.

In relation to insect chitosan in acetic acid, the behavior was similar to that observed for crustacean chitosan at both pH and inoculum size values. There was a decrease in the number of cells in comparison with the initial inoculum concentration. Nevertheless, according to the results that can be seen in Fig 2A and 2B, the antimicrobial activity of the crustacean chitosan is somewhat greater than showed by the insect chitosan.

### Antimicrobial effects of chitosan solutions on *S*. Typhimurium

Results of the evolution of *S*. Typhimurium in the different substrates with initial inoculation levels of 10^3^ CFU/mL and 10^6^ CFU/mL are shown in Fig 3A and 3B respectively.

As can be seen in Fig 3 for both inoculum sizes, there is a cell growth in the acetic acid substrate at pH values of 5 and 6.2. This means that acetic acid at these pH values of the study does not seem to have bacteriostatic or bactericidal effect against *Salmonella* Typhimurium.

The crustacean chitosan in acetic acid at pH 5 produced a reduction of the microbial load along the incubation period of 49 hours for the two initial inoculation levels. It appears that there was a synergistic effect among acetic acid and crustacean chitosan in acetic acid at this pH level. At pH 6.2, a slight decrease followed by a latency period was observed that lasted about 8 hours followed of an increase in the number of cells at hours 24 and 49 regardless of inoculum size.

The insect chitosan in acetic acid at pH 5 and with an inoculation level of 10^3^ CFU/mL produced a reduction in the number of viable cells at 24 hours of incubation period followed by a slight growth at 49 hours of incubation period. In the case of the inoculum level of 10^6^ CFU/mL a reduction of 3 log cycles was observed at 24 hours of incubation, but some growth appears to take place at 49 hours of incubation period manifested as a decrease of the log reduction cycles (Fig 3B).

At pH 6.2 a latency phase was observed that lasted until 8 hours of incubation, followed by an increase in the number of cells exceeding the level of initial inoculation. This behavior was similar for both initial inocula concentrations.

### Antimicrobial effects of chitosan solutions on *E*. *coli*, *L*. *monocytogenes* and *S*. Typhimurium after 8 hours of incubation

Tables 1–3 show the concentrations of microorganisms after 8 hour of incubation period at different pH values and inoculum concentrations for each microorganism considered in the study. The time of 8 hours of incubation was considered a good check point for comparison because it was the moment at which the stationary phase was reached with acetic acid at pH 6.2 used as control for comparison purpose.

**Table 1:** *E*. *coli* behaviour after 8 hours of incubation in different substrates (mean ± standard deviation).

Substrate	Log N/N_0_ (CFU/mL)			
	Inoculum size 10^3^		Inoculum size10^6^	
**Control (LB pH = 7)**	5.47± 0.075 ^A^		3.31±0.018 ^A^	
	**pH 5.0**	**pH 6.2**	**pH 5.0**	**pH 6.2**
**Acetic Acid Control**	-0.11±0.126 ^B^	4.39±0.041 ^B^	-0.09±0.004 ^B^	3.34±0.033 ^A^
**Crustacean chitosan**	-0.12±0.053 ^B^	-3.09±0.062 ^C^	-0.06±0.026 ^B^	-3.3±0.080 ^B^
**Insect chitosan**	-0.11±0.017 ^B^	-1.76±0.012 ^D^	-0.07±0.0236 ^B^	-1.46±0.045 ^C^

Figures with the same letter are not significantly different by columns. Different letters indicate significant differences (p value≤0.05). Negative figures mean inactivation and positive figures mean growth.

**Table 2:** *Listeria monocytogenes* behaviour after 8 hours of incubation in different substrates (mean ± standard deviation).

Substrate	Log N/N_0_ (CFU/mL)			
	Inoculum size 10^3^		Inoculum size10^6^	
**Control (TSB pH = 7)**	4.27±0.0269 ^A^		2.98±0.0170 ^A^	
	**pH 5.0**	**pH 6.2**	**pH 5.0**	**pH 6.2**
**Acetic Acid Control**	-0.37±0.023 ^B^	2.96±0.11 ^B^	-0.11±0.020 ^B^	2.73±0.046 ^B^
**Crustacean chitosan**	-1.07±0.036 ^C^	ND	-1.85±0.020 ^C^	-3.48±0.062 ^C^
**Insect chitosan**	-1.28±0.057 ^C^	-1.73±0.0698 ^C^	-0.013±0.049 ^B^	-3.78±0.136 ^D^

ND = Non Detectable. Figures with the same letter are not significantly different by columns. Different letters indicate significant differences (p value≤0.05).

Negative figures mean inactivation and positive figures mean growth.

**Table 3:** *Salmonella* Typhimurium behaviour after 8 hours of incubation in different substrates (mean ± standard deviation).

Substrate	Log N/N_0_ (CFU/mL)			
	Inoculum size 10^3^		Inoculum size10^6^	
**Control (TSB pH = 7)**	4.47±0.0566 ^A^		2.68±0.05 ^A^	
	**pH 5.0**	**pH 6.2**	**pH 5.0**	**pH 6.2**
**Acetic Acid Control**	0.95±0.0236 ^B^	4.42±0.07 ^A^	1.29±0.018 ^B^	2.12±0.046 ^B^
**Crustacean chitosan**	-1.28±0.045 ^D^	-0.57±0.012 ^C^	-1.51±0.0804 ^D^	-0.02±0.105 ^C^
**Insect chitosan acid**	-0.90±0.028 ^C^	-0.04±0.073 ^B^	-0.55±0.043 ^C^	-0.22±0.018 ^D^

Figures with the same letter are not significantly different by columns. Different letters indicate significant differences (p value≤0.05). Negative figures mean inactivation and positive figures mean growth.

Table 1 shows the *E*. *coli* microbial counts after 8h of incubation in blank control, acid control and chitosan treatments. According to the table, significant differences (p≤0.05) on bacterial counts were found between the blank control and the rests of substrates except for the inoculation level 10^6^ and pH 6.2. Besides, at pH 6.2 there were significant differences (p≤0.05) on counts among acetic acid, crustacean chitosan and insect chitosan substrates in the way that all chitosan treatments reduced the microbial load at pH 6.2 compared to acetic acid or blank controls (p≤0.05). In addition, crustacean chitosan showed a greater microbial reduction than insect chitosan.

As for *L*. *monocytogenes*, Table 2 shows bacterial counts after 8h of incubation in blank control and the studied substrates. Acetic acid control produced a small reduction of bacteria cells independent of inoculum size at pH 5 as compared with the blank control. Similarly to *E*. *coli* behaviour (Table 1), chitosan treatments at pH 6.2 showed greater *Listeria* cell reductions than at pH 5, being the maximum reduction achieved with insect chitosan at pH 6.2 for an inoculum size of 10^6^ CFU/mL (3.78 log reductions).

Table 3 shows the *S*. Typhimurium bacterial counts after 8h of incubation in blank control and the studied substrates. As can be seen, acetic acid control media resulted in an increase of microbial counts at all pH and inoculum concentrations. Regarding *Salmonella* cells exposed to chitosan, small reductions were observed in all cases, at 8 hour of incubation, showing significantly differences (p≤0.05) among them. According to the table, those reductions in *Salmonella* cells were dependent on the source of chitosan, pH and inoculation level. In general, crustacean chitosan was more effective against *Salmonella* than insect chitosan excepting at an inoculum size of 10^6^ CFU/mL and pH 6.2 (Table 3).

## Discussion

The effect of chitosan as an antimicrobial in agriculture and the food industry has been studied for some time ago. Allan et al. [36] already studied the effect of chitosan on *E*. *coli* and *Staphylococcus aureus* (*S*. *aureus*). However recently, and as a consequence of the use of minimum preservation procedures, the interest for the antimicrobial capability of this compound obtained from chitin has been accentuated. According to previous studies carried out by other authors, the antimicrobial activity of chitosan is influenced by several extrinsic and intrinsic factors (type of chitosan, molecular weight, degree of deacetylation, solvent and concentration) as well as some environmental factors such as microorganism specie, its physiological state, pH, temperature, ionic strength, metal ions, the presence of ethylenediaminetetraacetic acid (EDTA) or organic matter [37–40].

Chitosan is a biopolymer primarily commercially produced from crabs and shrimp residues. The physicochemical characteristics of chitosan influence its functional properties, which differ between species of crustaceans and methods of preparation [41]. In the present work, chitosan from crustaceans and insects have been studied. The objective was to compare the functionality as antimicrobials of commercial products of crustacean and insect chitosan. Results pointed out differences between the antimicrobial capacity of commercial crustacean chitosan and commercial insect chitosan. In general, at 49 hours of incubation period, the crustacean chitosan at pH 5.0 showed greater antimicrobial capacity than the insect chitosan at the same pH value. This behavior was mainly seen in the case of Salmonella where the crustacean chitosan produced more than 4 logarithmic reductions while the insect chitosan was bacteriostatic or produced around 1 logarithmic reduction. The same behavior is observed for *E*. *coli* although the differences in the antimicrobial activity are smaller than in the case of Salmonella. As indicated above, some previous studies have indicated that there may be differences between functional capacity and physical characteristics of chitosan from different crustaceans. This behavior could be more evident among chitosan from sources as diverse as crustaceans and insects.

The pH plays an important role in the antimicrobial capability of chitosan in such a way that the antimicrobial activity is inversely affected by the pH value; at low pH the chitosan seems more antimicrobial than at high pH values [38, 42–44].

Wang [45] conducted a study on the effect of different chitosan concentrations at two levels of pH 6.5 and 5.5 on different pathogenic microorganisms including *S*. Typhimurium, *E*. *coli* and *L*. *monocytogenes*. The author concluded that chitosan at pH 6.5 had a very weak effect on pathogenic microorganisms; in fact, there was no inhibition of *L*. *monocytogenes*. At pH 5.5 there was inhibition of the microorganisms tested between 24 and 72 hours of storage at 30°C. The author concluded that chitosan acts better at pH 5.5 than at pH 6.5. More recently, Gücükoğlu [46] also studied the antibacterial activity of chitosan of different molecular weights at various pH levels (pH 4, 4.5 and 5) on *L*. *monocytogenes* strains. Results indicated that the pH 5 showed also the greatest bacterial reduction effect at 24 hours of the incubation period excepting for two *L*. *monocytogenes* strains.

In the present work two pH levels were tested at a 0.15% (w/v) concentration of chitosan. After an incubation period of 8 hours, the antimicrobial effect was higher at pH 5.00 than at pH 6.2 for *Salmonella* Typhimurium, while for *E coli* and *Listeria* occurred the contrary, chitosan at pH 6.2 had a stronger antimicrobial effect than at pH 5.00. It appears that the effect of chitosan at both pH levels was microorganism dependant.

In our study, all microorganisms grew in the control at pH 7 reaching the stationary phase in many cases at 8 hours of the incubation period. The effect of control (acetic acid) at pH 5.0 appears to be microorganism dependent, it was bacteriostatic for *E*. *coli*, bactericidal for *L*. *monocytogenes* and in the case of *S*. Typhimurium there was a growth similar to that achieved in the control (acetic acid) at pH 6.2 after 49 hours of incubation. These results seem to indicate that the acetic acid control at pH 5.0 has an antimicrobial effect only on some microorganisms. Regarding the chitosan at the two pH levels, differences were observed regarding the control in acetic acid. Chitosan showed a clear antimicrobial activity, especially in *L*. *monocytogenes* at both pH values. Chitosan from both sources, crustacean and insect, at pH 5.0 were bacteriostatic or bactericidal for the three pathogenic microorganisms studied at the 49 hours of the incubation period, while for the same period of time, growth was observed on chitosan at pH 6.2, except on *L*. *monocytogenes* were still chitosan at pH 6.2 had a bactericidal effect. These results are in general in agreement with those obtained by Wang [45] who indicated that all chitosan concentrations tested at pH 5.5 had a greater antimicrobial effect than at pH 6.5 excepting some chitosan concentrations against *Samonella*. Gücükoğlu [46] also observed a general greater antimicrobial effect on the pH 5 tested on *L*. *monocytogenes* strains in spite of a different inoculum concentration was used in his study (2.0x10^5^ CFU/mL) compared with our study.

According to the results, it appears that the effect of chitosan was microorganism dependent; maybe differences in the membrane of the different microorganisms could affect the antimicrobial activity of chitosan. There are some hypotheses about the antimicrobial mechanism of chitosan. The ionic interactions between positive charges of amino groups and negative bacteria surface molecules in acid conditions alter the membrane permeability leading to cellular lysis [40, 47]. Another mechanism could be the interaction with essential nutrients for bacteria [48].

The bacterial inoculum size could also affect the bactericidal effect of chitosan [49]. Those authors indicated that after 4 hours of incubation for an inoculum size of 10^3^ cells/mL, all tested compounds proved to be bactericidal at any tested chitosan concentration; while for higher concentration of initial inoculum, 0.1% (w/v) of chitosan was only bacteriostatic; they also concluded that, independently of the inoculum level, 0.25% (w/v) of any chito-oligosaccharide mixture was sufficient to reduce the *E*. *coli* initial population by at least 3 log cycles. However, results of the present work, as indicated above in results section, are not conclusive regarding the effect of the inoculum size because it varied according to pH and microorganism specie; in consequence it is not possible to establish that in all cases there is a higher antimicrobial activity at one particular inoculum size.

## Conclusions

In the present study, both chitosans showed antimicrobial activity, although their effect depended on the pH and the microorganism studied. *L*. *monocytogenes* was the most sensitive at both pH values. Crustacean chitosan seemed more active than insect for the microorganisms studied and at the pHs tested, specially against *Salmonella*. However, more studies on the characterization of both chitosan compounds would be necessary to interpret these differences.

For the pHs studies, after 8h of incubation antimicrobial activity seems to be microorganisms’ dependent; the antimicrobial effect was higher at pH 5.00 than at pH 6.2 for *Salmonella*, while for *E*. *coli* and *Listeria* occurred the contrary. As for inoculum size, no clear conclusions can be drawn on the antimicrobial effect of the chitosan compounds studied.

## Supporting information

S1 Dataset Evolution of *Salmonella* Typhimurium, *Escherichia coli* O157:H7 and *Listeria monocytogenes* counts (CFU/mL) (Log N/N_0_) in acetic acid, crustacean chitosan and insect chitosan treatments with different initial inoculum concentrations at pH 5 and 6.2. (XLSX)
